# Development and Evaluation of a Novel Mucoadhesive Film Containing *Acmella oleracea* Extract for Oral Mucosa Topical Anesthesia

**DOI:** 10.1371/journal.pone.0162850

**Published:** 2016-09-14

**Authors:** Verônica Santana de Freitas-Blanco, Michelle Franz-Montan, Francisco Carlos Groppo, João Ernesto de Carvalho, Glyn Mara Figueira, Luciano Serpe, Ilza Maria Oliveira Sousa, Viviane Aparecida Guilherme Damasio, Lais Thiemi Yamane, Eneida de Paula, Rodney Alexandre Ferreira Rodrigues

**Affiliations:** 1 Department of Physiological Sciences, Piracicaba Dental School, University of Campinas, Piracicaba, Brazil; 2 Chemical, Biological and Agricultural Research Center (CPQBA), University of Campinas, Paulinia, Brazil; 3 Faculty of Pharmaceutical Sciences, University of Campinas, Campinas, Brazil; 4 Department of Biochemistry and Tissue Biology, Institute of Biology, University of Campinas, Campinas, Brazil; University of Pittsburgh, UNITED STATES

## Abstract

**Purpose:**

To develop an anesthetic mucoadhesive film containing *Acmella oleracea* (jambu) extract for topical use on oral mucosa.

**Methods:**

Ethanolic extracts from aerial parts of jambu were prepared by maceration. Pigment removal was obtained by adsorption with activated carbon. Three mucoadhesive films were developed using a film casting method: 10 or 20% of crude jambu extract (10% JB and 20% JB), and 10% of crude jambu extract treated with activated carbon (10% JBC). The mucoadhesive films were characterized regarding their uniformity, thickness, pH, and spilanthol content, and their stability was evaluated during 120 days. Gas chromatography was used to quantify the amount of spilanthol. *In vitro* tests determined the permeation of spilanthol across pig esophageal epithelium mucosa in Franz diffusion cells. Topical anesthetic efficacy was assessed *in vivo* using a tail flick test in mice.

**Results:**

The three mucoadhesive films showed physical stability and visual appearances suitable for use on oral mucosa. The permeation study revealed that the spilanthol from 10% JBC presented higher flux and permeability coefficient values, compared to 10% or 20% JB (p < 0.001). Moreover, 10% JBC showed better topical anesthetic efficacy than the other films (p < 0.01).

**Conclusion:**

Mucoadhesive film containing crude extract of jambu treated with activated carbon is a potential alternative for oral, topical use, encouraging future clinical studies.

## 1. Introduction

A significant fear-triggering agent during dental treatment is the perceived pain during anesthetic injections, which is a source for fear and anxiety that affects up to 30% of the global population [1–3]. In fact, such painful procedures have significantly inhibited patients from seeking appropriate dental treatment [4–7]. Local anesthetics cause a loss of sensitivity by acting on the nerve cell membrane, preventing the generation and conduction of nerve impulses [8]. Topical application of local anesthetics on oral mucosa generally produces superficial anesthesia, useful to reduce the pain during needle insertion or other minimally invasive dental procedures. To achieve an effective topical anesthesia, it is necessary that the topical anesthetic remains in the place of application for a minimum of two minutes [2].

Despite not being designed for oral use, the eutectic mixture of 2.5% lidocaine and 2.5% prilocaine (EMLA Cream^®^) is currently considered the most effective and potent topical anesthetic on oral mucosa [9–14]. Many studies have shown the superior topical anesthesia effectiveness of EMLA in dental procedures in comparison with commercial topical anesthetics, such as 5% lidocaine or 20% benzocaine. The high costs of commercial-synthetic topical anesthetics, which are economically viable only in small portions of the body, along with reports of severe neurotoxicity and cardiotoxicity induced by lidocaine and other topical anesthetics [15–17], justify the development of new substitutes. It is, therefore, relevant and timely to identify and develop new anesthetics suitable for minimizing oral pain associated with dental procedures [3, 18–20].

The constant interest in natural pharmaceuticals has led to great investigation in plant products. *Acmella oleracea* (L.) R.K. Jansen, also referred to as *Spilanthes acmella* L. Murray, is a native South American herb, being very common throughout Southeast Asia [21, 22]. Popularly known as jambu or paracress, it is traditionally used by the northern Brazilian population as a food spice and for the treatment of toothaches and other ailments affecting gums and throat.

Several compounds have been identified in jambu, such as β-caryophyllene, limonene, and thymol in the essential oil, along with vanillic acid, trans-ferulic acid, stigmasterol, β-sitosterol, rhamnogalacturonan, scopoletin and alkyl amides in extracts [23–27]. Spilanthol (synonym: affinin), an N-alkyl amide, is one of the bioactive compounds of jambu, being used as flavoring agent in soups, processed vegetables, condiments, chewing gum and dentifrices [28]. When ingested, it causes tingling, numbness and increased salivation [29, 30]. The high concentration of spilanthol found in this species is responsible for both analgesic and anti-inflammatory effects [31, 32]. Studies have evaluated the antinociceptive activity of jambu, and the proposed mechanism of action include the modulation or blocking of transient receptor potential channels subfamily V member 1 (TRPV1) and subfamily A member 1 (TRPA1) [33] and the increased release of gamma-aminobutyric acid [34].

The anesthetic activity of jambu was previously described [35, 36]. Jambu is also classified as safe (GRAS #3783) by the Flavor and Extract Manufacturers Association (FEMA) [37] and the European Food Safety Authority (EFSA) [38]. It presents low toxicity [33, 39, 40] and a widespread popular use. Considering the current lack of effective formulations for topical anesthesia on oral mucosa, jambu is a good candidate for topical anesthesia.

During the development of formulations described here, chitosan was used as a film-forming agent due to its biocompatibility, biodegradability, and non-toxicity. Furthermore, chitosan exhibits buccal mucoadhesive capacity, interacting with negatively-charged groups of the epithelium surface. These characteristics contributed to the selection of chitosan as the film-forming polymer in this study [41, 42].

The aim of the present study was to develop a mucoadhesive film for oral anesthetic application that could be used as a pre-anesthetic before injection in dental procedures.

## 2. Material and Methods

### 2.1 Materials

All the reagents and solvents used were analytical or chromatographic grade. The materials used were as follows: chitosan (deacetylation degree > 75%) (Sigma-Aldrich^®^, MO, USA); Transcutol^®^ (ethoxydiglycol) (Gattefossé^®^, Lyon, France); methylparaben, glacial acetic acid, ethanol, ethyl acetate, n-hexane, and methanol (Synth^®^, São Paulo, Brazil); activated carbon (specification type in patent number BR102014022486-6); diatomaceous earth (Celite^®^ 545) (Nuclear^®^, São Paulo, Brazil); eutectic mixture of 2.5% lidocaine and 2.5% prilocaine (5% EMLA^®^ cream—AstraZeneca^®^, São Paulo, Brazil); chromatography grade methanol (J.T. Baker^®^, PA, USA).

### 2.2 Production of the Crude Ethanolic Extract of Jambu

The plant material used in this study was obtained from the experimental field cultivation at the Chemical, Biological, and Agricultural Research Center (CPQBA), University of Campinas (UNICAMP), located in Paulinia (São Paulo State, Brazil; 22° 47' 52" S, 47° 6' 49" W). The identification was confirmed by Dr. John F. Pruski of Missouri Botanical Garden, USA. A voucher specimen is deposited in the Herbarium of UNICAMP, under catalog #181452. The aerial parts of the jambu were dried, milled, and extraction was performed under mechanical agitation with 95% ethanol (1:5 w/v) in a stainless steel tank for 1.5 h. The remaining residue was separated by filtration, and the extraction process was repeated two more times. The final material was filtered, combined, concentrated under vacuum, and then lyophilized until dryness, being used to determine the final dry mass by weighing, to calculate the yield value. The process yield was determined by the relation between the aerial material used and the final dry mass of the extract. The estimated concentration of spilanthol in the extract was 3%. It was stored in the refrigerator at 8°C until treatment with activated carbon.

#### 2.2.1 Extract treatment with activated carbon

**Fig 1:**
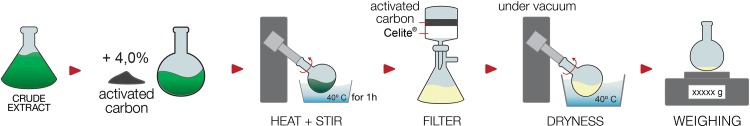
Illustration of the crude extract treatment process with activated carbon.

The extraction procedure was repeated to confirm the reproducibility of the process, but without the drying step. The extracts obtained were then combined and filtered, being added 4% (w/w) of activated carbon (Fig 1). The mixture was stirred and treated using a particulate activated carbon [43]. The extract was concentrated under vacuum, lyophilized to dryness, and stored in the refrigerator at 8°C until further use.

### 2.3 Spilanthol Analysis

Analysis of spilanthol in the extract and mucoadhesive films was performed using a gas chromatograph coupled to a mass spectrometer (GC-MS, Agilent^®^ 5890 Series II with Agilent^®^ 5970 EI 70eV mass selective detector). The GC was equipped with a fused silica WCOT column (Agilent^®^ HP5-MS, 30 m x 0.25 mm x 0.25 μm). The analysis conditions were: injector temperature of 220°C, detector temperature of 250°C, temperature program 60–240°C at 3°C/min, sample injection using split mode with 1:40 ratio, helium as carrier gas (0.7 bar, 1 mL/min).

A calibration curve was prepared for the analytical determination of spilanthol, using spilanthol isolated with a centrifugal partition chromatograph (Model 250-SCPC/Spot Prep II^®^, Armen, Saint-Ave, France) equipped with a quaternary pump, UV/Vis detector, and a fraction collector. The purity of 95.1% was found using GC-FID with area normalization. A stock solution of known concentration was prepared, followed by dilutions performed in volumetric flasks. The limit of detection (0.26 μg.mL^-1^ / LOD) and limit of quantification (0.86 μg.mL^-1^ / LOQ) was calculated based on the standard deviation of the response and the slope using three independent analytical curves. LOD and LOQ were calculated as 3 and 10 times, respectively, the standard deviation of the response/slope of the calibration curve [44].

### 2.4 Preparation of Mucoadhesive Films Containing *Acmella Oleracea*

**Fig 2:**
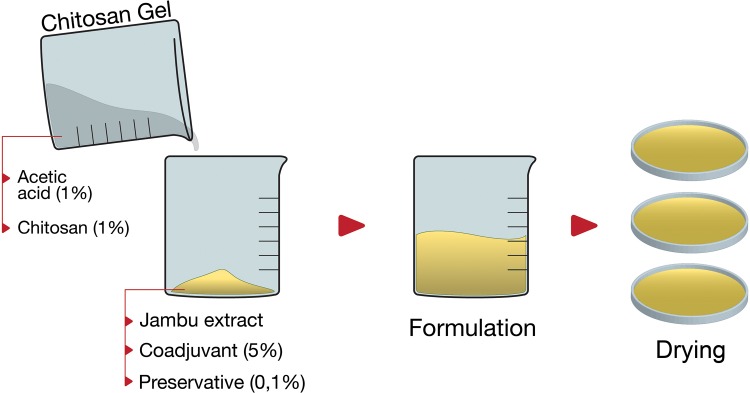
Illustration of the mucoadhesive film production.

The mucoadhesive films were prepared using the casting technique resulting in a gel (Fig 2). Briefly, chitosan (1 g) was dissolved in 1% (v/v) acetic acid solution, with the aid of a mechanical homogenizer, and the solution was poured into molds (polystyrene Petri dishes). The final step in the mucoadhesive films production was carried out in a gravity convection drying oven at 40°C for 30 h. Jambu extracts at concentrations of 10% or 20%, with 0.1% of methylparaben and 5% of transcutol were mixed with chitosan gel prepared previously according to patent number BR102014022486-6 [43].

The mucoadhesive films used for the *in vitro* and *in vivo* assays were prepared with 10% crude extract of jambu (10% JB), 20% crude extract of jambu (20% JB), or 10% crude extract of jambu treated with 4% of activated carbon (10% JBC).

### 2.5 Mucoadhesive Films Characterization

Physicochemical evaluation of the mucoadhesive films considered the uniformity of weight and thickness, pH, spilanthol content, and stability.

The mucoadhesive films were cut with a circular punch (15 mm diameter), the mass was measured using an analytical balance (Mettler-Toledo^®^, São Paulo, Brazil), and the thickness was measured using a digital pachymeter (Model Cal II, Tesa^®^, Renens, Switzerland).

The physicochemical stability of the mucoadhesive films was evaluated by packing the materials in waterproof aluminum-lined plastic containers with hermetic closures, and storing them at 40 ± 1°C for 120 days in a climate chamber with no humidity control. Samples were analyzed at 0 and 120 days, considering their appearance, pH, and spilanthol content. The tests were performed in triplicate.

The pH was measured in accordance with the Brazilian Pharmacopeia method [45]. Briefly, deionized water was added to the mucoadhesive films at 1% (w/w), the mixture was ultrasonicated for 2 min, and the pH was measured at room temperature with a pH-meter previously calibrated at pH 7 and 4.

For the determination of spilanthol content, a known mass of mucoadhesive films was added to 1% (m/m) methanol and ultrasonicated for 10 min, followed by filtration through a 0.45 μm membrane filter. The spilanthol quantification was performed according to the analytical conditions described in Section 2.3.

### 2.6 *In vitro* Permeation Studies

*In vitro* permeation studies were carried out using Franz-type vertical diffusion cells with permeation area of 0.6 cm^2^ and receptor compartment volume of 4.2 mL. The assays were performed using pig esophagus epithelium obtained from a local slaughterhouse (Frigar Abatedouro Industria e Comercio de Conservas Ltda–ME, located in Sousas-Pedreira Road, Sao Paulo St Brazil, 22° 51' 50.2" S, 46° 59' 59.3" W), according to the method described by Diaz del Consuelo (2005) [46]. The use of pig esophageal epithelium was demonstrated to be an equivalent barrier model to buccal epithelium, since it has similar histological characteristics, permeability, and epithelium composition [46–48]. Moreover, esophageal mucosa has some advantages including larger surface area, absence of damage caused by mastication and easy preparation [46].

Briefly, within 2 h of slaughter, the pig esophageal mucosa was carefully separated from the surrounding tissue with a scalpel. Mucosae with any visual damage at the surface were discarded. The epithelium was separated from the connective tissue after immersion in deionized water at 60°C for 2 min and it was used immediately. This temperature was demonstrated to be unable to alter integrity or permeability of the tissue [46, 49, 50]. The epithelium was placed over a 0.45 μm cellulose filter, with the connective side of the tissue facing the membrane filter, due to its fragility [46, 47]. The membrane filter avoids epithelium damage, without altering drug permeation. In addition, it reduces the release of impurities from the epithelium to the receptor solution.

The mucoadhesive film, epithelium, and membrane filter were clamped between the donor and receptor compartments. Saline-methanol (70:30, v/v) solution was used in the receptor compartment in order to maintain the sink conditions. The experiment was performed at 37°C during 5 h, under magnetic stirring (400 rpm). Samples (300 μL) were periodically withdrawn from the receptor compartment and immediately replaced by the same volume of solution, taking account of dilution effects. The samples were transferred to chromatography vials and stored in a refrigerator until GC-MS analysis for quantification of spilanthol.

The cumulative amount of spilanthol transported across esophagus epithelium per area unit was plotted along time. The active steady-state flux (*J*) across the barrier was calculated from the slope of the linear portion of the curve. The *lag time* was obtained from the intercept on the time axis, and the permeability coefficient was calculated according to the following equation [46, 50, 51]:

$$J=P\times C_{d}$$

where *J* (μg.cm^-2^.h^-1^) is the spilanthol flux across the epithelium, *P* (cm.h^-1^) is the permeability coefficient, and *C*_*d*_ is the spilanthol concentration in the donor compartment (μg/cm^3^). All experiments were conducted six times.

### 2.7 *In vivo* Anesthetic Efficacy

#### 2.7.1 Animals

Male Swiss mice (25–40 g) from the Multidisciplinary Center of Biological Investigation of Laboratory Animals (CEMIB–UNICAMP) were maintained at 25 ± 2°C under light/dark cycles of 12 h and were kept in their cages with water and food *ad libitum* for at least 7 days before the experiments. The trials were conducted after approval by the Animal Ethics Committee of UNICAMP (protocol #2851–1) and in accordance with the Principles of Laboratory Animal Care (NIH publication #85–23, 1985). The mice were divided into groups of 5 to 6 animals, and each animal was used only once in the experiment.

#### 2.7.2 Tail-flick test

The topical anesthetic efficacy of the mucoadhesive films containing jambu extract was evaluated using the tail-flick test, as previously described by de Araujo et al. (2010) [51], which slightly modified Grant et al. (1993) method [52]. Briefly, the animal was placed in an acrylic restraint while maintaining freely the distal portion of the tail (10 cm). The time required for tail removal (latency) was considered as the aversive response to the heat generated by an incandescent lamp (55°C). The baseline was recorded for each animal before the start of the experiment, and only those with baselines below 4 s were considered suitable. The maximum time for contact of the tail with the heat source was set at 10 s (cut-off value) to avoid thermal injury. The three mucoadhesive films prepared were compared to EMLA, used as a positive control (150 mg/animal, corresponding to 7.5 mg of anesthetic) and a negative control (chitosan-bioadhesive without the jambu extract). EMLA was chosen due to its efficacy to reduce pain during needle insertion [53–56] and during local anesthetic injection [57–59].

The mucoadhesive films and EMLA cream were applied 2 cm from the tail base, with the aid of an adhesive tape, for 2 min. The tested substances were then removed, and the nociceptive stimulus was applied to the same region. Measurements were performed immediately after mucoadhesive removal and then every 15 min until the animal returned to its baseline pain response. After use of the animals in the tail-flick test, they were euthanized by cervical dislocation. The duration of analgesia was defined as the increase in the time required for withdrawal of the tail, which was at least 50% higher than the baseline value observed. The data were expressed as the percentage of the maximum possible effect (MPE, in minutes), using the following relation:

**%MPE = [(test latency–baseline latency / cut-off time–baseline latency) x 100]**, being the area under the curve (AUC) recorded for each experimental group [60].

## 3. Statistical Analysis

The *in vitro* permeation data were expressed as a percentage or mean ± SD and subjected to one-way analysis of variance (one-way ANOVA) with the Holm-Sidak *post hoc* test. Analgesia duration was analyzed by Kruskal-Wallis/SNK tests. Correlation between the *in vivo* efficacy and the *in vitro* data was performed using the Pearson’s correlation test. All analyses were performed using GraphPad Prism 6.0^®^ (GraphPad Software, Inc., USA), considering a significance level of 5%.

## 4. Results

### 4.1 Preparation of Extracts

The ethanolic crude extract of jambu yielded 7.7 ± 0.08%, while the crude extract treated with 4% activated carbon yielded 4.0 ± 0.03%, both on a dry basis. The yield decrease observed for crude extract with 4% activated carbon did not affect the amount of spilanthol. In fact, the spilanthol concentration was increased by using this procedure, being the greenish pigments removed.

### 4.2 Characterization of the Mucoadhesive Films

Table 1 shows the mean (±SD) values for thickness, mass, pH and spilanthol content obtained during the mucoadhesive films stability study.

**Table 1:** Physicochemical parameters (Mean±SD) of the mucoadhesive films and spilanthol extracted from mucoadhesive films.

Extracts	Thickness(in mm)(n = 7)	Mass (in g)(n = 7)	pH (n = 3)		Spilantholcontent (mg/g)	
			Day 0	Day 120	Day 0	Day 120
**10% crudeextract**	0.45 ± 0.02	0.13 ± 0.01	5.3 ± 0.03	5.4 ± 0.24	17.6 ± 1.41	16.7 ± 0.21
**20% crudeextract**	0.53 ± 0.01	0.14 ± 0.01	5.1 ± 0.02	5.1 ± 0.05	35.9 ± 5.59	36.2 ± 2.62
**10% extract +4% activated carbon**	0.52 ± 0.01	0.14 ± 0.01	4.9 ± 0.02	5.3 ± 0.05	22.9 ± 3.39	25.0 ± 0.85

Day 0 = the day of production; Day 120 = the maximum period in the climate chamber.

After 120 days of storage at 40°C, no significant degradation of spilanthol was verified in the mucoadhesive films. The pH remained stable, with minimal changes.

### 4.3 *In vitro* Permeation Studies

**Fig 3:**
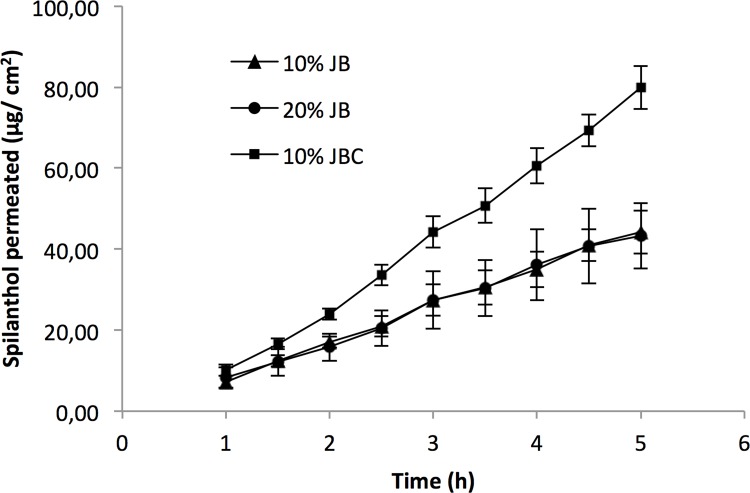
Permeation profiles across pig esophagus mucosa of spilanthol from mucoadhesive films applied under finite dose conditions (mean ± SD, n = 6). 10% JB: mucoadhesive containing 10% of dry crude extract; 20% JB: mucoadhesive containing 20% of dry crude extract; 10% JBC: mucoadhesive containing 10% of dry extract treated with activated carbon (4%).

Fig 3 shows the spilanthol permeation profiles of the different mucoadhesive films across pig esophagus mucosa. The linear regression analysis showed higher (p < 0.0001) spilanthol permeation for 10% JBC mucoadhesive film in comparison with both 10% JB and 20% JB films, which did not differ from each other (p = 0.474). Despite the 20% JB film was two-fold more concentrated than 10% JB, the spilanthol permeation profiles of both were similar.

The permeation parameters (flux, lag time, and permeability coefficient) obtained for the experiment illustrated in Fig 3 are provided in Table 2.

**Table 2:** Parameters for the permeation (5 h) through pig esophageal mucosa, under finite dose conditions, of spilanthol applied using the three mucoadhesive films tested (mean ± SEM; n = 6; JB: dry crude extract; JBC: dry crude extract treated with 4% of activated carbon).

Mucoadhesive films(Spilanthol concentration)	Flux(μg.cm^2^.h^-1^)	Lag time(h)	Permeability coefficient(×10^-3^cm.h^-1^)
10% JB (2.37 mg)	9.18 ± 1.19^a^	0.16 ± 0.04^a^	3.86 ± 0.50^a^
20% JB (5.13 mg)	9.52 ± 1.97^a^	0.25 ± 0.06^a^	1.84 ± 0.38^b^
10% JBC (3.14 mg)	17.70 ± 4.36^b^	0.58 ± 0.15^b^	5.17 ± 1.39^c^

Each permeation parameter was analyzed separately by ANOVA/Holm-Sidak. Different letters mean significant statistical differences (p<0.05) among the mucoadhesive films.

The 10% JBC mucoadhesive film presented higher flux of spilanthol across pig esophageal mucosa when compared to both 10% JB (p = 0.0003) and 20% JB (p = 0.0004), which did not differ from each other (p = 0.8351). However, 10% JBC mucoadhesive film showed a significantly longer lag time (p < 0.0001) than the other films. It also presented the highest permeability coefficient (p < 0.01), while 10% JB film showed higher permeability coefficient than 20% JB (p = 0.0032).

### 4.4 Tail-Flick Test

**Fig 4:**
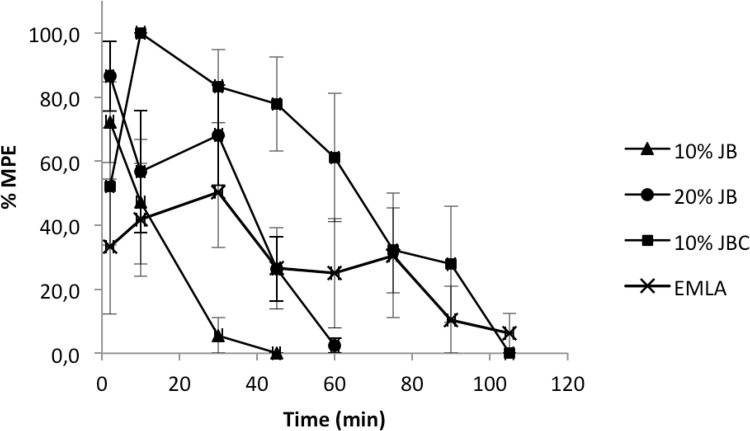
Percentage of maximum possible effect (%MPE) values for the different formulations. Data are presented as mean ± SEM (n = 6). %MPE = [(test latency–baseline latency / cut-off time–baseline latency) x 100].

Fig 4 shows the tail-flick test results, presented as the percentage of maximum possible effect (%MPE).

The 10% JBC mucoadhesive film presented significantly higher (p < 0.001) area under the curve (AUC_0-105min_) than the other formulations (Table 3).

**Table 3:** Duration of analgesia and AUC values for the three tested mucoadhesives and EMLA^®^.

Mucoadhesive films	Analgesia duration in min Median (1^st^– 3^rd^ quartiles)	AUC_(0–105 min)_mean ± SD
**10% JB (n = 6)**	6.0 (2–10)^a^	230.4 ± 46.4 ^a^
**20% JB (n = 6)**	30 (30–30)^ab^	417.3 ± 59.6^b^
**10% JBC (n = 6)**	60 (33.8–86.2)^b^	754.3 ± 131.2^c^
**EMLA**^**®**^ **(n = 6)**	75 (75–75)^b^	569.8 ± 160.0^d^

JB: dry crude extract; JBC: dry crude extract treated with 4% of activated carbon; Analgesia duration was analyzed by Kruskal-Wallis/SNK tests; AUC was analyzed by ANOVA/Holm-Sidak tests. Each parameter was analyzed separately. Different letters mean statistically significant differences among the films.

As expected, no antinociceptive effect was observed in animals treated with chitosan mucoadhesive films without dry crude extract of jambu (negative controls).

### 4.5 *In vitro/In vivo* Correlation

**Fig 5:**
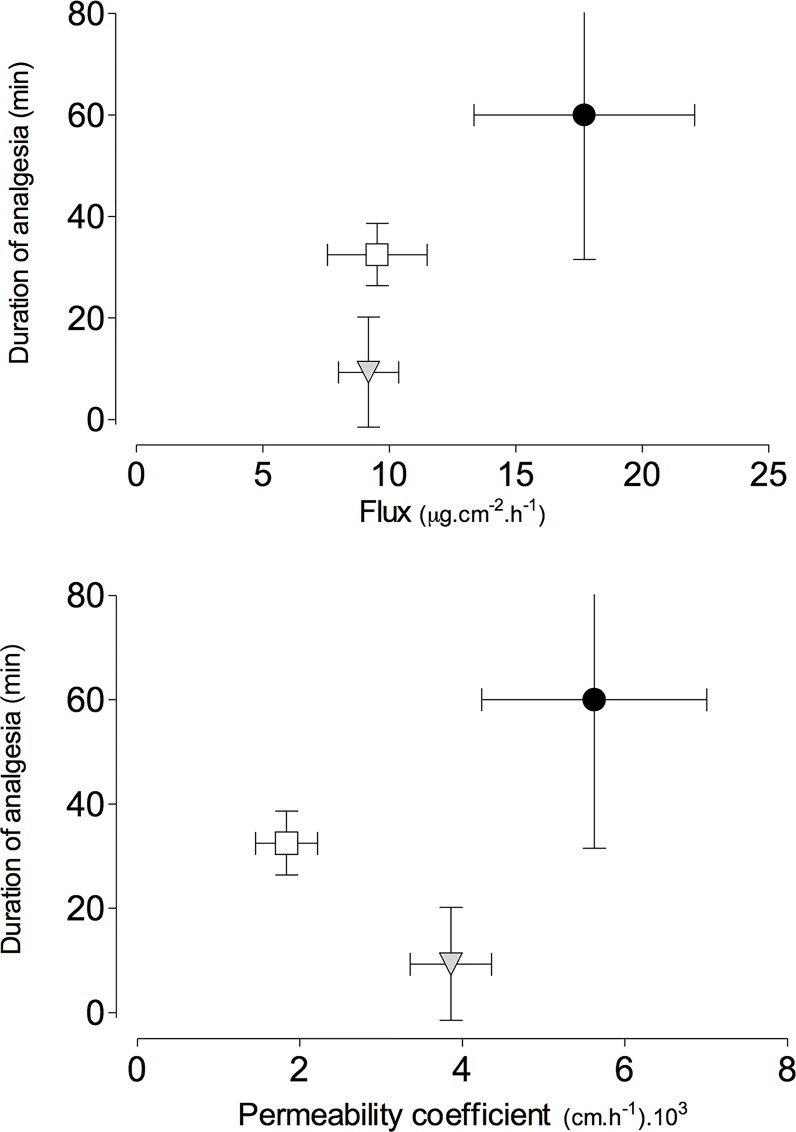
Pearson coefficient values (mean ± SD) for correlation between the studied factors and analgesia duration. Black circle: 10% JBC; white square: 20% JB; gray triangle: 10% JB.

Fig 5 shows the Pearson coefficient (r) values for the correlation analysis between the permeation parameters (flux and permeability coefficient) and duration of analgesia in the tail-flick test. A strong correlation (r = 0.89) was found between analgesia duration and flux, but there was no correlation with the permeability coefficient (r = 0.7).

## 5. Discussion

Spilanthol presents good stability in ethanolic extracts [61]. In addition, ethanol is relatively safe, and it presents low toxicity and cost, being widely used in the extraction of natural products[62, 63]]. Therefore, ethanol was the solvent chosen to produce jambu extracts in the present study. The crude jambu extraction yield obtained in this study is comparable to values reported in other studies [64, 65].

Activated carbon has been used to remove chlorophyll or other compounds that might interfere with colorimetric assays, and to isolate bioactive compounds [66, 67]. Activated carbon includes a range of substances with a high degree of porosity and surface area around 800–1500 m^2^/g [68]. It has been also used in many applications, such as removal of color, taste, odor, and other impurities in water [69], and in food processing [70], due to its excellent absorbance capacity, biocompatibility, and lack of toxicity. In the present study, the use of activated carbon was effective to remove some crude extract pigments, such as chlorophyll. The 4% concentration used was found to be the optimal percentage in a preliminary study. Despite the reduction in the mass of extract, a relative increase in the spilanthol concentration was observed.

The mucoadhesive films showed homogeneity of thickness and mass. The average thickness (0.5 mm) of the mucoadhesive films produced in this study was similar to that observed by Aksungur et al. (2004) [71], who developed chitosan bioadhesives with nystatin to treat oral mucositis. Storage at 40°C for 120 days did not affect the physical integrity, pH, or spilanthol content of the adhesives, indicating the good stability of the formulations.

A vertical Franz diffusion cell is commonly used to evaluate the permeation of compounds through the skin or mucosa [72]. The selection of porcine esophageal mucosa as the barrier in this study was based on the similar histological characteristics of this tissue and oral mucosa, such as the presence of non-keratinized stratified squamous epithelium, large surface area and typically intact tissue, favoring the tissue handling during the Franz cells assembly [73].

The *in vitro* permeation of spilanthol in porcine oral mucosa has been investigated elsewhere using Franz-type vertical diffusion cells. Boonen et al. (2010) [74] evaluated two formulations of oral gel (Indolphar^®^ and Buccaldol^®^), as well as the pure ethanolic extract of jambu with propylene glycol, using a porcine buccal mucosa (426 ± 10 μm) as permeation barrier.

The differences verified in the concentration of the active compound and the composition of the formulation could lead to different permeation profiles. de Araujo et al. (2010) [51] evaluated multiple formulations of benzocaine, observed almost overlapping permeation profiles in an infinite-dose design and a dramatically different behavior in a finite-dose condition. Boonen et al. (2010) [74] and in the present study, similar permeation profiles of spilanthol were obtained for the films, despite the substantial differences in spilanthol concentration and film composition.

All the formulations evaluated in the current work permeated the pig esophageal epithelium mucosa at higher flux rates than those described by Boonen et al. (2010) [74]. This difference could be explained by the thinner barrier used (mucosa epithelium rather than the thicker dermatomized mucosa). In addition, higher spilanthol concentration (at least 10 times greater) was used in our study, causing an increased flux of the active agent, in agreement with Fick’s first law of diffusion.

Nevertheless, the flux is also influenced by the composition of the formulation [75]. Formulations with different concentrations of the same drug may exhibit the same flux, as observed here for the 20% JB mucoadhesive film, which showed a flux very similar with the 10% JB film, despite containing twice the spilanthol concentration. However, the 10% JBC mucoadhesive film presented a flux almost two-fold higher than both 10% JB and 20% JB films, despite the intermediate amount of spilanthol.

These features can also be observed for the lag time and permeability coefficient, with formulations containing the same concentration of the active agent, but different composition on excipients, showing different solubility of the active compound during transport across the barrier [51]. In the present case, the lag time and permeability coefficient were higher for the 10% JBC mucoadhesive film, compared to the other films. Similar findings were reported by Fang et al. (2008) [76] who showed the addition of menthol and ethanol to tetracaine gels doubling the flux and the lag time. The authors suggested that penetration enhancers acted to increase the lag time due to slower distribution in the skin. Another possibility is the presence of a higher concentration of terpenes in the 10% JBC mucoadhesive film, which are typically observed in *Acmella oleracea* extracts [30], and cause an increase in the lag time and in the flux of various drugs [77–79].

A longer lag time, however, should not be confused with a delay in the onset of the anesthetic effect *in vivo*, as demonstrated by Fang et al. (2008) [76]. They observed that a topical tetracaine formulation with the greatest lag time *in vitro* showed the shortest anesthetic onset time in volunteers. The same profile was described by Woolfson et al. (1998) [80], also using tetracaine gel, where the lag time *in vitro* did not affect the *in vivo* onset of anesthesia. Similarly, in the present study, the lag time did not have any effect on the beginning of analgesia *in vivo*.

Consequently, we hypothesize that the removal of pigments by the activated carbon treatment was responsible for the better *in vitro* permeation results achieved with the 10% JBC mucoadhesive. However, this hypothesis should be confirmed in further phytochemical studies. To the best of our knowledge, there are no studies regarding the effect of activated carbon on the phytochemical composition of extracts from plants.

The tail-flick test has been demonstrated to be effective in the antinociceptive activity evaluation of different topical formulations and mucoadhesive films [51, 81–83], and this assay was chosen to evaluate the effectiveness of the mucoadhesive films in the present study.

The antinociceptive effect of jambu has been assessed previously using the tail-flick test. Chakraborty et al. (2004) [39] demonstrated the analgesic activity of its aqueous extract after intraperitoneal administration in albino rats at doses of 100, 200, and 400 mg/kg. Barman et al. (2009) [84] reported the analgesic effect of an ethanolic extract of jambu after subcutaneous administration to albino rats at a dose of 100 mg/kg. Interestingly, the antinociceptive effect was observed with only two minutes of application of the mucoadhesive film on the tail of the animals, showing a rapid onset of action, as desired for dental procedures. The spilanthol concentrations in the mucoadhesive films (10% JB: 2.37 mg; 20% JB: 5.13 mg; 10% JBC: 3.14 mg) were lower than the concentration of anesthetic in the positive control (EMLA^®^: 7.5 mg lidocaine/prilocaine).

The correlation between *in vivo* effectiveness and *in vitro* drug permeation has been investigated in several studies [51, 75, 85–88]. Since *in vivo* studies are usually more expensive, involve ethical aspects, and are more time consuming than *in vitro* studies, efforts to standardize the conditions of *in vitro* experiments and to achieve better replication during the evaluation of different formulations have been performed [89]. The observation of a strong correlation between the permeability coefficient and the duration of analgesia was in agreement with other previous study [51], which observed the analgesia duration increasing with the amount of benzocaine permeated across pig ear skin *in vitro*.

A strong correlation was also found between the flux and the duration of analgesia, which could be explained by greater amounts of spilanthol reaching the receptors responsible for the analgesic effect. In a previous study, Franz-Montan et al. (2013) [75] reported a strong correlation between the flux of benzocaine across pig esophageal epithelium and anesthetic efficacy in volunteers, although the duration of anesthesia was not reported.

The molecular properties of spilanthol could also contribute to the results observed in the present study since it meets all the requirements proposed by Lipinski et al. in 1997 [90], which are known as "rule of five". The five requirements are set of parameters established to predict whether a compound will or will not be orally bioavailable, being widely used to find new molecules for drug development. These five requirements or rules are: 1) octanol-water partition coefficient (*log P*) less than five; 2) molecular polar surface area (PSA) of 60 to 70 Å; 3) molecular weight should not exceed 500 g/mol; 4) less than 10 groups accepting hydrogen atoms to form hydrogen bonds and 5) less than 5 groups in the molecule donating hydrogen atoms to the hydrogen bonds. Spilanthol has *log P* of 3.4, PSA of 29.1, a molecular weight of 221 g/mol, two atoms of hydrogen acceptors, and one hydrogen donor atom. Thus, it is likely to be permeable through membranes and it could be easily absorbed.

## 6. Conclusions

An orally mucoadhesive film based on chitosan appears to be a promising option for use as a vehicle for the topical application of an anesthetic based on jambu ethanolic extract. The ethnopharmacological uses of this plant species, its use in the culinary area, its safety, and its low toxicity indicate that it can provide an alternative to the topical anesthetic formulations currently available in dentistry.

The optimum formulation developed showed a high degree of *in vitro* permeation and an *in vivo* anesthetic effect similar to EMLA, used as a gold standard topical anesthetic, indicating its potential as an alternative to the topical anesthetics currently found on the market.

## Supporting Information

S1 Fig Spilanthol permeated (μg/cm^2^). (DOCX)

S2 Fig Percentage of maximum possible effect (%MPE) values for the different formulations. (DOCX)

S1 Table Physicochemical parameters of the mucoadhesive films and spilanthol extracted from mucoadhesive films. (DOCX)

S2 Table Parameters for the permeation (5 h) through pig esophageal mucosa, under finite dose conditions, of spilanthol applied using the three mucoadhesive films tested. (DOCX)

S3 Table Duration of analgesia and AUC values for the three tested mucoadhesives and EMLA^®^. (DOCX)
