# Supplementary material for: Development and Evaluation of a Novel Mucoadhesive Film Containing Acmella oleracea Extract for Oral Mucosa Topical Anesthesia
Source: PLoS One. 2016 Sep 14;11(9):e0162850. doi: 10.1371/journal.pone.0162850 (PMC5023158; doi:10.1371/journal.pone.0162850)
Supplement: S1 Fig — (DOCX) [file pone.0162850.s001.docx]

**Supporting Information**

**S1 Fig 3 - Spilanthol permeated (µg/cm^2^)**

|  |  | Time (hours) | | | | | | | | | |
| --- | --- | --- | --- | --- | --- | --- | --- | --- | --- | --- | --- |
|  |  | 0.5 | 1 | 1.5 | 2 | 2.5 | 3 | 3.5 | 4 | 4.5 | 5 |
| 10% crude extract | Cell 1 | 4.8 | 7.8 | 12.7 | 17.5 | 22 | 27.7 | 31.4 | 36.1 | 41.1 | 44.9 |
|  | Cell 2 | 6.5 | 9.5 | 13.5 | 18.8 | 23.8 | 33.2 | 36.2 | 40.6 | 46 | 53.2 |
|  | Cell 3 | 4.2 | 6.2 | 14.3 | 18.2 | 23.5 | 29.9 | 33.9 | 38.9 | 43.6 | 47 |
|  | Cell 4 | 3 | 6.1 | 10.7 | 16.2 | 18.1 | 22 | 24.3 | 28.9 | 0 | 38.9 |
|  | Cell 5 | 4.5 | 6.4 | 11.7 | 15.1 | 18.2 | 25.9 | 28.1 | 31.9 | 36.4 | 39.8 |
|  | Cell 6 | 4.9 | 0 | 11.5 | 16.7 | 20.1 | 25.8 | 29.4 | 33.5 | 38 | 41.7 |
|  |  |  |  |  |  |  |  |  |  |  |  |
| 20% crude extract | Cell 1 | 7.4 | 8.5 | 11 | 14.1 | 18.3 | 0 | 27.4 | 31.4 | 36.9 | 44 |
|  | Cell 2 | 1.8 | 5.8 | 11.6 | 13.1 | 18.4 | 24.6 | 27.4 | 0 | 36.3 | 41.2 |
|  | Cell 3 | 6.9 | 5.8 | 7.8 | 13.5 | 14.9 | 19.7 | 21.2 | 25.2 | 29.8 | 33.9 |
|  | Cell 4 | 9.6 | 10.9 | 15.2 | 18.4 | 23.8 | 29 | 36.4 | 42.3 | 47.7 | 56 |
|  | Cell 5 | 10.7 | 7.3 | 10.5 | 14.2 | 20.4 | 0 | 29.3 | 34.5 | 38.5 | 41.2 |
|  | Cell 6 | 11.1 | 11.6 | 17.9 | 21.6 | 27.1 | 36.3 | 40.6 | 47.4 | 55.5 | 101.7 |
|  |  |  |  |  |  |  |  |  |  |  |  |
| 10% extract +  4% activated carbon | Cell 1 | 6 | 6.7 | 10.5 | 18.4 | 26.7 | 0 | 39.6 | 48.4 | 60.7 | 72.7 |
|  | Cell 2 | 0 | 0 | 12.5 | 17 | 24.9 | 30.4 | 35.8 | 42.3 | 48.2 | 59.5 |
|  | Cell 3 | 5.5 | 8.1 | 13 | 17.1 | 24.7 | 28 | 36.3 | 46.3 | 50.4 | 57.7 |
|  | Cell 4 | 0 | 10.1 | 20.2 | 31.8 | 39.7 | 52.3 | 57.4 | 67.4 | 81.5 | 93.5 |
|  | Cell 5 | 9.7 | 15.9 | 24.5 | 33.5 | 44.2 | 55.1 | 68.4 | 78.1 | 87.4 | 100.6 |
|  | Cell 6 | 7.2 | 10.1 | 19.4 | 26 | 41.2 | 55.5 | 67 | 81.4 | 87.6 | 95.3 |
