# Supplementary material for: Development and Evaluation of a Novel Mucoadhesive Film Containing Acmella oleracea Extract for Oral Mucosa Topical Anesthesia
Source: PLoS One. 2016 Sep 14;11(9):e0162850. doi: 10.1371/journal.pone.0162850 (PMC5023158; doi:10.1371/journal.pone.0162850)
Supplement: S2 Fig — (DOCX) [file pone.0162850.s002.docx]

**Supporting Information**

**S2 Fig 4 -** **Percentage of maximum possible effect (%MPE) values for the different formulations.**

|  | % EMP (mean ± SD) | | | |
| --- | --- | --- | --- | --- |
| Time (in min) | 10% crude extract | 20% crude extract | 10% extract +  4% activated carbon | EMLA |
| 2 | 72.2 (± 12.7) | 86.5 (± 10.9) | 52.1 (± 18.4) | 33.3 (± 21.1) |
| 10 | 47.2 (± 19.4) | 56.7 (± 19.1) | 100 (± 0) | 41.7 (± 17.6) |
| 30 | 5.6 (± 5.6) | 68.2 (± 15.7) | 83.3 (± 11.4) | 50.4 (± 17.4) |
| 45 |  | 26.4 (± 10.1) | 77.8 (± 14.7) | 26.5 (± 12.6) |
| 60 |  | 2.4 (± 2.4) | 61.1 (± 20) | 25 (± 17.1) |
| 75 |  |  | 32.1 (± 13.4) | 30.6 (± 19.4) |
| 90 |  |  | 27.8 (± 18.1) | 10.4 (± 10.4) |
| 105 |  |  |  | 6.3 (± 6.3) |
