# Supplementary material for: Development and Evaluation of a Novel Mucoadhesive Film Containing Acmella oleracea Extract for Oral Mucosa Topical Anesthesia
Source: PLoS One. 2016 Sep 14;11(9):e0162850. doi: 10.1371/journal.pone.0162850 (PMC5023158; doi:10.1371/journal.pone.0162850)
Supplement: S1 Table — (DOCX) [file pone.0162850.s003.docx]

**Supporting Information**

**S1 Table 1-** **Physicochemical parameters of the mucoadhesive films and spilanthol extracted from mucoadhesive films.**

|  | Thickness (mm) | | |  | Mass (g) | | |
| --- | --- | --- | --- | --- | --- | --- | --- |
| n | 10% crude extract | 20% crude extract | 10% extract + 4% activated carbon |  | 10% crude extract | 20% crude extract | 10% extract + 4% activated carbon |
| 1 | 0.44 | 0.53 | 0.53 |  | 0.1167 | 0.1427 | 0.1348 |
| 2 | 0.44 | 0.53 | 0.53 |  | 0.118 | 0.1435 | 0.1446 |
| 3 | 0.44 | 0.54 | 0.52 |  | 0.1193 | 0.1404 | 0.1472 |
| 4 | 0.47 | 0.53 | 0.53 |  | 0.1259 | 0.133 | 0.1449 |
| 5 | 0.44 | 0.51 | 0.51 |  | 0.1328 | 0.1359 | 0.1523 |
| 6 | 0.43 | 0.51 | 0.52 |  | 0.1403 | 0.1519 | 0.1333 |
| 7 | 0.48 | 0.54 | 0.53 |  | 0.1449 | 0.1437 | 0.1327 |
| Mean | 0.45 | 0.53 | 0.52 |  | 0.13 | 0.14 | 0.14 |
| SD | 0.02 | 0.01 | 0.01 |  | 0.01 | 0.01 | 0.01 |

|  | pH | | | | | |
| --- | --- | --- | --- | --- | --- | --- |
|  | 10% crude extract | | 20% crude extract | | 10% extract + 4% activated carbon | |
| n | Day 0 | Day 120 | Day 0 | Day 120 | Day 0 | Day 120 |
| 1 | 5.32 | 5.55 | 5.08 | 5.1 | 4.95 | 5.32 |
| 2 | 5.27 | 5.60 | 5.11 | 5.2 | 4.92 | 5.28 |
| 3 | 5.29 | 5.16 | 5.08 | 5.13 | 4.92 | 5.38 |
| Mean | 5.3 | 5.4 | 5.1 | 5.1 | 4.9 | 5.3 |
| SD | 0.03 | 0.24 | 0.02 | 0.05 | 0.02 | 0.05 |

|  | Spilanthol content (mg/g) | | | | | |
| --- | --- | --- | --- | --- | --- | --- |
|  | 10% crude extract | | 20% crude extract | | 10% extract + 4% activated carbon | |
| n | Day 0 | Day 120 | Day 0 | Day 120 | Day 0 | Day 120 |
| 1 | 18.6 | 16.8 | 31.9 | 38 | 20.5 | 24.4 |
| 2 | 16.6 | 16.5 | 39.8 | 34.3 | 25.3 | 25.6 |
| Mean | 17.6 | 16.7 | 35.9 | 36.2 | 22.9 | 25.0 |
| SD | 1.41 | 0.21 | 5.59 | 2.62 | 3.39 | 0.85 |
