# Supplementary material for: Development and Evaluation of a Novel Mucoadhesive Film Containing Acmella oleracea Extract for Oral Mucosa Topical Anesthesia
Source: PLoS One. 2016 Sep 14;11(9):e0162850. doi: 10.1371/journal.pone.0162850 (PMC5023158; doi:10.1371/journal.pone.0162850)
Supplement: S2 Table — (DOCX) [file pone.0162850.s004.docx]

**Supporting Information**

**S2 Table 2-** **Parameters for the permeation (5 h) through pig esophageal mucosa, under finite dose conditions, of spilanthol applied using the three mucoadhesive films tested (mean ± SEM; n = 6; JB: dry crude extract; JBC: dry crude extract treated with 4% of activated carbon).**

|  | Flux (μg.cm2.h-1) | | | Lag time (h) | | | Permeability coefficient  (×10^-3^cm.h-1) | | |
| --- | --- | --- | --- | --- | --- | --- | --- | --- | --- |
| n | 10% crude extract | 20% crude extract | 10% extract  + 4% activated carbon | 10% crude extract | 20% crude extract | 10% extract  + 4% activated carbon | 10% crude extract | 20% crude extract | 10% extract  + 4% activated carbon |
| 1 | 9.34 | 8.78 | 16.259 | 0.13 | 0.27 | 0.82 | 3.93 | 1.69 | 5.17 |
| 2 | 10.94 | 8.75 | 12.92 | 0.21 | 0.33 | 0.63 | 4.6 | 1.69 | 4.1 |
| 3 | 10.09 | 6.95 | 12.69 | 0.19 | 0.25 | 0.53 | 4.24 | 1.34 | 4.03 |
| 4 | 7.71 | 11.29 | 20.225 | 0.13 | 0.25 | 0.51 | 3.24 | 2.18 | 6.43 |
| 5 | 8.37 | 8.98 | 21.36 | 0.17 | 0.27 | 0.36 | 3.52 | 1.73 | 6.79 |
| 6 | 8.61 | 12.4 | 22.73 | 0.11 | 0.15 | 0.64 | 3.62 | 2.39 | 7.22 |
| Mean | 9.18 | 9.53 | 17.7 | 0.16 | 0.25 | 0.58 | 3.86 | 1.84 | 5.62 |
| SD | 1.19 | 1.97 | 4.36 | 0.04 | 0.06 | 0.15 | 0.5 | 0.38 | 1.39 |
