# Supplementary material for: Development and Evaluation of a Novel Mucoadhesive Film Containing Acmella oleracea Extract for Oral Mucosa Topical Anesthesia
Source: PLoS One. 2016 Sep 14;11(9):e0162850. doi: 10.1371/journal.pone.0162850 (PMC5023158; doi:10.1371/journal.pone.0162850)
Supplement: S3 Table — (DOCX) [file pone.0162850.s005.docx]

**Supporting Information**

**S3 Table 3 - Duration of analgesia and AUC values for the three tested mucoadhesives and EMLA^®^.**

|  | Analgesia duration (min) | | | |
| --- | --- | --- | --- | --- |
| n | 10% crude extract | 20% crude extract | 10% extract +  4% activated carbon | EMLA |
| 1 | 10 | 30 | 90 | 75 |
| 2 | 10 | 30 | 90 | 75 |
| 3 | 30 | 45 | 45 | 90 |
| 4 | 2 | 30 | 30 | 75 |
| 5 | 2 | 30 | 75 | 75 |
| 6 | 2 | 30 | 30 | 10 |
| Median | 6 | 30 | 60 | 75 |
| 1st quartile | 2 | 30 | 33.8 | 75 |
| 3rd quartile | 10 | 30 | 86.2 | 75 |

|  | AUC(0-105 min) | | | |
| --- | --- | --- | --- | --- |
| n | 10% crude extract | 20% crude extract | 10% extract +  4% activated carbon | EMLA |
| 1 | 230 | 362.5 | 940 | 517 |
| 2 | 240 | 374 | 838 | 797 |
| 3 | 217.5 | 412.5 | 696 | 742.5 |
| 4 | 184 | 374.5 | 593 | 496 |
| 5 | 196 | 505 | 812.5 | 412 |
| 6 | 315 | 475 | 646 | 454 |
| Mean | 230.4 | 417.3 | 754.3 | 569.8 |
| SD | 46.4 | 59.6 | 131.2 | 160 |
